# Effects of Nutritional Supplementation on Fatigue, and Autonomic and Immune Dysfunction in Patients with End-Stage Renal Disease: A Randomized, Double-Blind, Placebo-Controlled, Multicenter Trial

**DOI:** 10.1371/journal.pone.0119578

**Published:** 2015-03-06

**Authors:** Sanae Fukuda, Hidenori Koyama, Kazuhiro Kondo, Hisako Fujii, Yoshinobu Hirayama, Tsutomu Tabata, Mikio Okamura, Tomoyuki Yamakawa, Shigeki Okada, Sumio Hirata, Hiroshi Kiyama, Osami Kajimoto, Yasuyoshi Watanabe, Masaaki Inaba, Yoshiki Nishizawa

**Affiliations:** 1 University of Welfare Sciences, Kasiwara, Osaka, 582-0026, Japan; 2 RIKEN Center for Life Science Technologies, Kobe, Hyogo, 650-0047, Japan; 3 Department of Physiology, Osaka City University Graduate School of Medicine, Osaka, 545-8585, Japan; 4 Department of Metabolism, Endocrinology and Molecular Medicine, Osaka City University Graduate School of Medicine, Osaka, 545-8585, Japan; 5 Department of Internal Medicine, Division of Diabetes, Endocrinology and Metabolism, Hyogo College of Medicine, Nishinomiya, Hyogo, 663-8501, Japan; 6 Department of Virology, The Jikei University School of Medicine, Tokyo, 105-8461, Japan; 7 Center for Drug & Food Clinical Evaluation, Osaka City University Hospital, Osaka, 540-0051, Japan; 8 College of Pharmaceutical Sciences, Ritsumeikan University, Kusatsu, 525-8577, Japan; 9 Inoue Hospital, Suita, 564-0053, Japan; 10 Ohno Memorial Hospital, Osaka, 550-0015, Japan; 11 Shirasagi Hospital, Osaka, 546-0002, Japan; 12 Okada Clinic, Osaka, 543-0056, Japan; 13 Kumamoto University School of Pharmacy, Kumamoto, 862-0973, Japan; 14 Nagoya University School of Medicine, Nagoya, 466-8550, Japan; 15 Department of Medical Science on Fatigue, Osaka City University Graduate School of Medicine, Osaka, 545-8585, Japan; University of Sevilla, SPAIN

## Abstract

**Background:**

Fatigue is a predictor of cardiovascular events in patients with end-stage renal disease (ESRD) undergoing hemodialysis treatment. We hypothesized that multinutritional support would improve quality of life, fatigue symptoms, and potential quantitative measures including endocrine, immune and autonomic functions in patients with ESRD undergoing hemodialysis.

**Methods:**

Two hundred and two hemodialysis patients were randomly assigned to receive active treatment (containing vitamin B1, vitamin B2, niacin, vitamin B6, vitamin B12, folic acid, vitamin C, carnitine, coenzyme Q10, naïve galacto-oligosaccharide, and zinc) or placebo after each dialysis session for 12 weeks. The patients and attending physicians were blinded to the treatment, and 172 patients (86 in each group) completed the study. Fatigue was evaluated via fatigue questionnaire at 0, 4, and 12 weeks. To assess human herpes virus (HHV) 6 and 7 reactivation, numbers of viral DNA copies were determined in saliva by polymerase chain reaction at weeks 0 and 12. Autonomic function was determined via measurement of beat-to-beat variation by using acceleration plethysmography.

**Results:**

Clinical characteristics, changes in fatigue, quality of life score, endocrine functions, and laboratory data did not differ significantly between the two groups. Several parameters of heart rate variability significantly increased after nutritional treatment compared to placebo. Nutritional drink for 12 weeks significantly suppressed HHV7 DNA copy numbers. Similarly, HHV6 DNA copy numbers tended to be decreased by treatment but without reaching statistical significance.

**Conclusions:**

Nutritional supplementation may modulate immune and autonomic dysfunction in ESRD patients undergoing hemodialysis.

## Introduction

Fatigue is a frequent symptom in patients with end-stage renal disease (ESRD) undergoing maintenance dialysis. The prevalence of fatigue ranges from 60% to as high as 97% in ESRD patients on long-term dialysis [1]. We have recently shown that the high fatigue score predicts cardiovascular events in hemodialysis patients [2], suggesting that fatigue is an important risk factor in high-risk populations, such as ESRD. Moreover, quality of life (QOL) score and poor sleep quality are independently associated with clinical outcomes, including prospective hospitalization and mortality in these populations [3,4].

The potential underlying mechanisms linking fatigue and QOL with cardiovascular events may include autonomic and immune dysfunction. Sudden cardiac death may be related to poor autonomic function with a significant decrease in heart rate variability (HRV) [5]. In ESRD patients, HRV is decreased, and may be isolated to either the parasympathetic limb or combined parasympathetic and sympathetic damage [6]. Several studies have demonstrated that decreased HRV can be predictive of left ventricular hypertrophy and mortality in ESRD patients [7]. In our recent cross-sectional study, fatigue and impaired QOL were associated with impaired HRV in hemodialysis patients [8]. Furthermore, immunological dysfunction, resulting in an infection, inflammation, or both, is closely associated with poor clinical outcomes in ESRD patients [9]. The major etiology of morbidity and mortality in ESRD patients is infection. Uremia is associated with a state of immunological dysfunction that may contribute to occurrence of cardiovascular disease [10]. Human herpes virus (HHV)6 and 7 reactivation in saliva is associated with inflammatory cytokine production, and thereby represents a decline in immune function [11]. Active infection with HHV6 and HHV7 may trigger and perpetuate chronic fatigue syndrome in a subset of patients [12]. Indeed, HHV6-induced chronic viral activation in patients with central nervous system dysfunction has been reported [13]. However, to the best of our knowledge, there are no previous studies on viral reactivation in ESRD patients, with the exception of one report on transplantation status [14].

Owing to the complexity of fatigue, a multidisciplinary approach should be adopted to reduce fatigue in hemodialysis patients. In turn, this approach may be beneficial in preventing cardiovascular events, as well as improving QOL. A few trials have suggested that improvements in nutritional status via intravenous l-carnitine [15]or growth hormone treatment [16,17] may improve fatigue and QOL of dialysis patients. However, there are no randomized controlled trials on the efficacy of direct nutritional supplementation on fatigue and its related measurements. In this pilot trial, we examined the effects of nutritional supplementation on the symptoms of fatigue, QOL, and endocrine, autonomic and immune dysfunction in ESRD patients undergoing hemodialysis.

## Methods

The protocol for this trial and supporting CONSORT checklist are available as supporting information; see S1 CONSORT Checklist and S1 Protocol.

### Study design

The study was approved by The Ethics Committee of Osaka City University Graduate School of Medicine (Approval No. 1232) in November 2007 and individual hospitals, and conducted in accordance with the Declaration of Helsinki. All participants gave written informed consented to participate in the study before enrollment. We registered the study to UMIN* 000001055: The efficacy of the nutrient supplement, AMP01, on fatigue in hemodialysis patients, 01/03/2008 (the protocol is shown in the S1 protocol).

The trial was conducted at four dialysis centers in the Osaka district in Japan between March and August 2008. We defined this study as a pilot study and determined the target sample size at 200, because little is known about the effect of nutritional support on fatigue or fatigue-related measures in ESRD patients. The enrollment was closed when the target sample size was reached.

The safety of the intervention and scientific integrity of the study were supervised by an independent data and safety monitoring board located at the Center for Drug & Food Clinical Evaluation, Osaka City University Hospital, Osaka, Japan (coordinating center). Patients aged 30–70 years being treated for ESRD for at least 1 year with afternoon hemodialysis three times a week were eligible to participate. The exclusion criteria were as follows: (1) active malignant tumor; (2) pregnancy; or (3) lactation. Patients who had been taking vitamins before recruitment were included after a washout phase of at least 2 weeks.

This was a multicenter, randomized, placebo-controlled, parallel-group study. Randomization by means of a computer-generated random number table (1:1) was to either the nutritional drink, or matching placebo in accordance with the minimization method with three factors (sex, age, each of four dialysis center); one drink was taken by patients after each dialysis session under the supervision of a nurse. One bottle of “AMP01” or placebo was to be administered three times a week just after hemodialysis sessions for 12 weeks.

Stratification was conducted by sex, age (30–50, 51–59, and 60–69) and each of four dialysis centers. Originally assigned code numbers were kept in closed envelopes within the coordinating center. All study investigators, medical staff, statistician and participants were blinded to the randomization procedure and treatment assignments.

The nutritional drink was 50 ml of liquid containing 10 mg vitamin B1, 1.8 mg vitamin B2, 15 mg niacin, 10 mg vitamin B6, 30 μg vitamin B12, 0.5 mg folic acid, 60 mg vitamin C, 500 mg carnitine, 30 mg coenzyme Q10 (CoQ10), 5 g naïve galacto-oligosaccharide, and 8 mg zinc, whereas the placebo drinks consisted of a similarly flavored colored water (50 ml). Nutritional and placebo drinks were visibly indistinguishable.

The treatment period was 12 weeks, with a midpoint assessment after 4 weeks from individual enrollment.

### Study outcomes

The outcome of the study was the changes in the acute and chronic fatigue scales, QOL, levels of serum adrenocorticotropic hormone (ACTH), cortisol and α-melanocyte stimulating hormone (α-MSH), HHV6 and 7, and autonomic function, determined by HRV. We did not determine the primary and secondary outcomes because of the nature of the pilot study.

The demographic and clinical data were collected upon study entry. Nonfasting blood was collected prior to the first dialysis session (after the weekend) at weeks 0, 4, and 12 of the trial for the determination of blood counts and clinical chemical, lipid, and inflammation parameters. As potential biomarkers of fatigue, HHV6 and 7 in saliva, serum ACTH was measured by immunoradiometric assay, and serum cortisol and α-MSH were measured by radioimmunoassay [18] at weeks 0, 4, and 12. DNA samples were extracted from 400 μl saliva with a Virus Mini Kit v2.0 (Qiagen、Tokoo, Japan) were stored at −20°C until further use. HHV6 and HHV7 DNA levels were quantified via real-time polymerase chain reaction (PCR), which was carried out in a PE Applied Biosystems Sequence Detector 7300. After 30 s at 95°C, samples were subjected to 50 cycles of 95C for 5 s, followed by 60°C for 31 s, to amplify HHV6, HHV7 and internal controls. The cycle threshold value was defined as the first cycle number at which fluorescence was greater than that of the threshold. Each sample was run in duplicate, and the mean of the two values was defined as the sample copy number. DNA fragments of HHV6 were first amplified by PCR using Taq1: GACAATCACATGCCTGGATAATG as the forward primer and Taq2: TGTAAGCGTGTGGTAATGGACTAA as the reverse primer, and then detected with the probe H6S: FAM-AGCAGCTGGCGAAAAGTGCTGTGC-TAMRA. DNA fragments of HHV7 were first amplified by PCR using HHV-7HHV-7U37F3: CGGAAGTCACTGGAGTAATGAC as the forward primer and HHV-7U37R2: CCAATCCTTCCGAAACCGAT as the reverse primer, and then detected with the probe HHV-7U37 TM2: CCTCGCAGATTGCTTGTTGGCCATG. Each PCR contained 5.0 μl DNA, 25.0 μl [P remix Ex Taq (Perfect Real Time); Takara Bio, RR039B), 1.0 μl ROX, 16.85 μl water, 10 μM primer, and 100 μM of each primer. Inter- intra- assay in HHV6 and 7 were normally within 10% and if each value was over 25%, the sample was re-tested.

All laboratory analyses, except HHV6 and HHV7, and α-MSH, were performed in the central laboratory of Mitsubishi Chemical Medience Corporation (Tokyo, Japan).

Fatigue and QOL were measured at weeks 0, 4 and 12 of the trial. The questionnaire associated with chronic fatigue was recently described [2]. Participants were asked to rate how often they experienced the symptoms of fatigue in a recent week using a Likert scale (0–4). Eight factors were calculated as a part of the principal factor analysis with a promax rotation. They were as follows: fatigue, anxiety and depression, loss of attention and memory, pain, overwork, autonomic imbalance, sleep problems, and infection. The acute fatigue scale associated with hemodialysis was evaluated with a visual analog scale (VAS). QOL was evaluated via the Kidney Disease Quality of Life questionnaire short form (KDQOL-SF) [19]. Sexual function in kidney-disease-related QOL was omitted from the analyses, because the response rate was <30%.

To analyze HRV, beat-to-beat variation of each participant was measured and analyzed with an acceleration plethysmography (APG) system (ARTETD; Umedica, Osaka, Japan), as previously described, with slight modifications [20]. APG waves were recorded from one finger in the sitting position, while fully clothed. An APG wave is the second differential wave of a pulse wave, which stabilizes the baseline of the waves and allows for an accurate measurement of rate intervals (consecutive a-wave intervals). To assess autonomic function, the spontaneous beat-to-beat variations, according to time domains [standard deviations (SDs) of all normal a-wave intervals (CVa-a%)] and frequency domains [low frequency (LF) and high frequency (HF) power, and LF/HF ratio], were determined [21]. For the analyses of frequency domains, the consecutive a-a intervals were analyzed by the maximum entropy method, where the power spectra at 0.02–0.15 Hz corresponded to LF, and 0.15–0.5 Hz to HF. The resolution of the spectrum was set at 0.001 Hz. From the power spectra, the LF and HF components were presented as ms^2^ [21].

### Statistical analysis

The baseline characteristics of the patients in the two treatment arms are expressed as absolutes, percentages, and means ± SD, where appropriate. Differences in the two arms were determined via the χ^2^ test, *t* test and Mann–Whitney test (LF/HF, CVa-a%, cortisol, ACTH, αMSH, and HHV6 and 7). At each time point, the VAS score, eight fatigue-related factors, and KDQOL-SF were tested via mixed model for repeated measurements to determine the effects of time and treatment (placebo vs. nutritional drink). The number of copies of HHV6 and HHV7 were assessed via the Wilcoxon rank sum test, according to the nutritional group. All of these analyses were performed on SPSS version 22 (IBM, Tokyo, Japan).

## Results

### Study population

**Fig 1:**
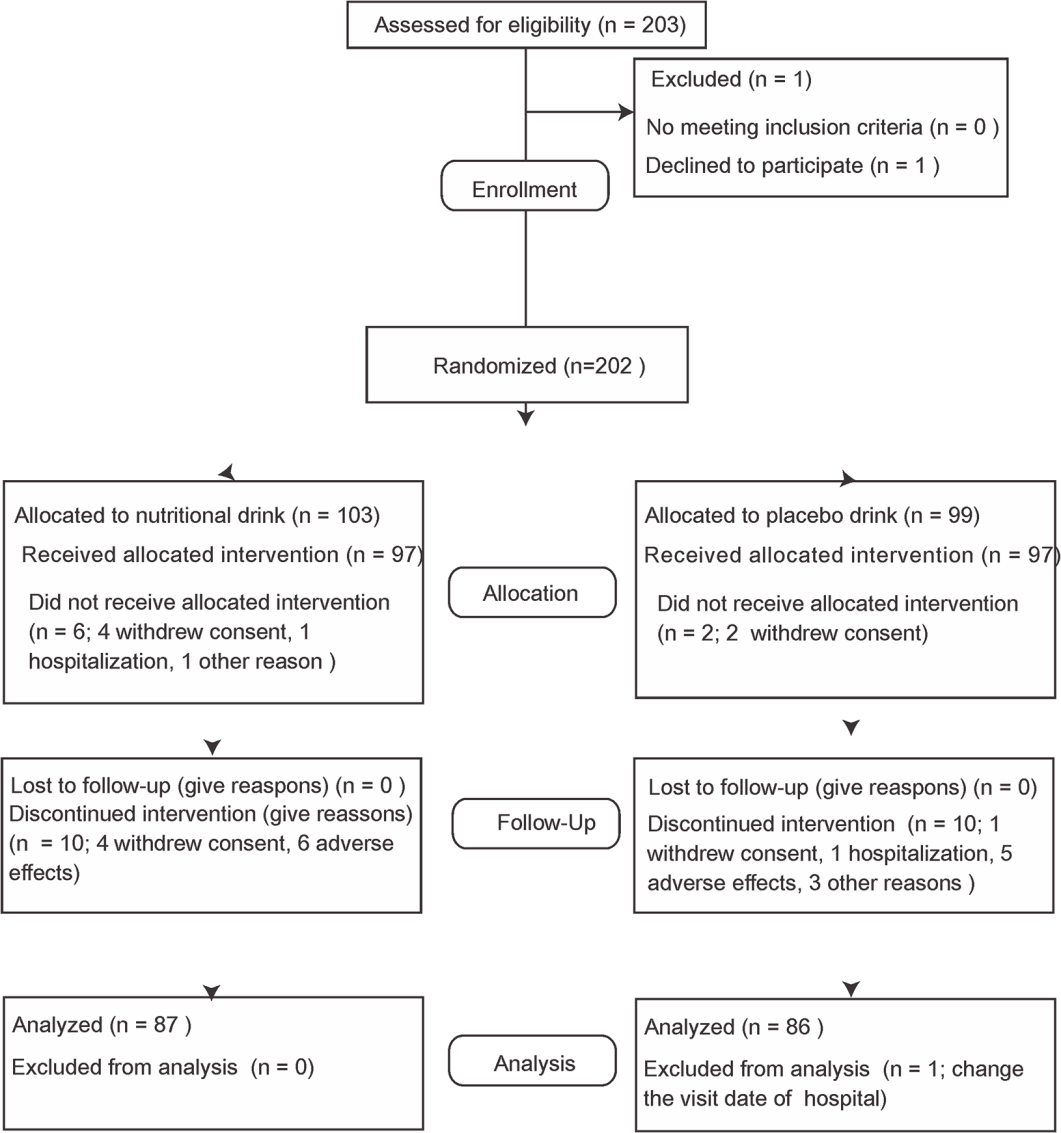
Flowchart of enrollment of randomized control study.

Fig. 1 shows participant flow from screening to last assessment. A total of 203 participants were assessed for eligibility. One participant withdrew consent before the randomization and a total of 202 patients [Inoue Hospital, Suita, Japan (n = 72); Ohno Memorial Hospital, Osaka, Japan (n = 54); Okada Clinic, Osaka, Japan (n = 31); Shirasagi Hospital, Osaka, Japan (n = 46)] were included in the trial and were randomly assigned to one of the two treatment arms. Of the 202 participants, six in the nutritional drink group and two in the placebo group did not receive allocation. Four participants in the nutritional group and two in the placebo group did not receive allocation because they withdrew consent. Two participants in the nutritional drink group did not receive allocation because of hospitalization or changing the time of dialysis from afternoon to morning. Ten participants in each group discontinued intervention (in the nutritional group, 4 withdrew consent and 6 experienced adverse effects; in the placebo group, 1 withdrew consent, 1 was hospitalized, 5 experienced adverse effects, 2 changed the time of dialysis from afternoon to morning, and 1 had unknown reasons). Finally, 68 patients in Inoue Hospital, 43 in Ohno Memorial Hospital, 24 in Okada Clinic, and 39 in Shirasagi hospital completed the intervention. One patient was excluded from the final analysis because of changing the hospital visit date from a weekday to the weekend. Originally assigned code numbers for intervention allocation were open after participants for analysis were finalized.

### Baseline clinical and demographic characteristics

The clinical and demographic characteristics of the patients are presented in Table 1. The baseline characteristics in the placebo and nutritional drink groups were well balanced, and there were no significant differences between any of the characteristics, except plasma glucose levels.

**Table 1:** Clinical characteristics of the study participants.

Variables	Allocated groups		
	Placebo drink	Nutritional drink	*P*-value
	N = 87	N = 87	
Age (years)	56.2±8.9	55.6±10.0	0.66
Gender (% male)	82.3	81.1	0.85
Current smoking (%)	34.8	30.8	0.85
Alcohol intake (%)	42.0	44.0	0.88
Dialysis vintage (years)	11.0±7.74	10.6±8.26	0.78
Single dialysis (h)	4.13±0.35	4.09±0.40	0.48
Working (days a month)	17.4±18.3	14.2±13.1	0.27
Diabetes (%)	24.5	24.5	
History of coronary artery disease (%)	44.9	45.6	0.90
History of cerebrovascular disease (%)	8.16	6.80	0.79
Systolic blood pressure (mmHg)	152.9±22.6	150.6±23.7	0.50
Diastolic blood pressure (mmHg)	78.3±13.3	75.8±11.4	0.20
Hemoglobin (g/dL)	10.2±1.00	10.3±0.88	0.60
CRP (mg/dL)	0.20±0.35	0.27±0.52	0.30
Albumin (g/dL)	3.88±0.32	3.90±0.31	0.71
Glucose (mg/dL)	103.5±35.6	120.2±63.0	0.03
Non-HDL cholesterol (mg/dL)	172.1±38.4	176.8±37.3	0.39
HDL cholesterol (mg/dL)	48.0±14.0	46.9±15.2	0.62

Conversion factors for units: hemoglobin in g/dL to g/L ×10, CRP in mg/dL to ng/L ×100, albumin in g/dL to g/L ×10, glucose in mg/dL to mmol/L ×0.0551, non-HDL cholesterol and HDL cholesterol in ng/dL to mmol/L ×0.02586.

All variables were tested via two-tailed *t* test, except gender, current smoking, alcohol intake, diabetes and history of diseases. These variables were tested by χ^2^ square test. CRP, C-reactive protein; HDL, high-density lipoprotein.

### Changes in QOL and fatigue symptoms

The baseline level of QOL and fatigue symptoms in the placebo and nutritional drink groups showed no significant differences. The effects of nutritional supplementation on the scores for acute or chronic fatigue, and QOL, are presented in Table 2. Acute fatigue, as determined by the VAS score, was not significantly affected by time, or time and treatment. Of the eight fatigue-related factors, fatigue, loss of attention and memory, and overwork were significantly decreased according to time, but not according to both time and treatment. These findings suggest that these factors were also significantly improved in the placebo group. Similarly, three factors for general QOL, specifically, role-physical, bodily pain, and role-emotional, and symptoms of the kidney-disease-specific QOL, specifically, effects of kidney disease, burden of kidney disease, and cognitive function, significantly improved over time, again independent of placebo or nutritional drink. Thus, subjective factors, such as fatigue and QOL symptoms, appear to be markedly influenced by the placebo effect.

**Table 2:** Changes in fatigue and QOL scores throughout the clinical trial.

	Allocated groups							
	Placebo drink			Nutritional drink			*P*- value (time)	*P*-value (time and treatment)
	Week 0	Week 4	Week 12	Week 0	Week 4	Week 12		
Fatigue (VAS)								
after dialysis	43.1±26.2	43.5±26.3	43.6±26.6	43.1±26.3	42.1±25.0	40.3±25.4	0.64	0.83
bed time	34.5±25.2	35.2±26.0	36.1±26.0	38.6±26.1	36.9±25.4	34.7±25.3	0.64	0.44
next morning	26.6±22.7	29.5±26.3	30.2±25.8	26.5±23.2	27.7±22.1	29.7±23.5	0.21	0.97
next night	23.9±23.9	22.7±21.9	24.5±23.1	21.8±24.9	23.4±22.9	27.0±25.2	0.37	0.40
Fatigue score								
fatigue	6.21±4.62	5.57±4.51	5.31±4.52	6.28±4.68	5.47±4.28	5.59±4.56	0.003	0.93
anxiety & depression	3.83±4.02	3.83±4.00	3.75±4.39	4.92±4.26	4.37±4.37	4.39±4.22	0.12	0.34
loss of attention & memory	6.15±4.97	5.64±4.72	5.37±4.88	5.87±4.21	5.34±4.21	5.23±4.37	0.002	0.83
pain	5.15±4.34	4.70±3.98	4.28±4.22	5.24±4.36	4.89±4.25	4.93±4.28	0.03	0.84
overwork	4.28±5.43	3.39±4.16	3.35±4.31	3.82±4.15	3.01±3.63	2.88±3.32	0.003	0.91
autonomic disturbances	2.87±3.65	2.88±3.84	2.75±3.76	3.07±3.84	3.13±3.99	3.23±3.53	0.91	0.84
sleep problems	4.75±4.57	4.16±4.15	4.22±4.05	4.09±4.54	3.92±3.75	3.98±3.77	0.25	0.44
infection	1.28±2.48	1.34±2.35	1.47±2.57	1.67±3.28	1.85±3.66	1.98±3.39	0.44	0.96
KDQOL (SF36)								
Physical functioning	70.7±23.6	72.6±24.2	71.9±24.3	76.0±20.7	76.9±21.0	78.5±19.1	0.11	0.36
Role physical	77.0±36.4	85.0±29.9	79.0±34.0	70.2±35.7	79.8±32.9	84.1±27.6	0.003	0.24
Bodily pain	72.6±20.0	77.0±20.4	77.8±21.2	74.0±23.4	76.3±21.3	79.6±20.8	0.004	0.88
General health	42.7±19.4	45.1±20.7	43.8±21.0	45.1±18.6	46.9±18.9	48.5±16.6	0.26	0.66
Vitality	56.8±20.3	58.6±20.4	58.5±20.5	57.2±20.2	59.9±21.4	62.2±22.4	0.08	0.66
Social functioning	79.7±24.4	80.9±23.0	83.1±23.3	82.6±20.8	86.6±18.0	84.5±19.9	0.81	0.38
Role emotional	77.3±36.5	80.7±36.3	84.0±31.8	73.0±38.1	84.3±32.4	83.0±31.1	0.003	0.87
Mental health	67.8±19.3	70.8±19.8	70.2±20.4	70.2±17.3	70.2±17.9	71.9±20.4	0.17	0.45
(kidney disease specific)								
Symptoms/Problems	80.3±13.9	81.3±14.3	84.0±11.6	81.8±12.7	83.8±10.8	84.2±13.1	<0.001	0.43
Effect of kidney disease	73.2±21.8	76.5±22.1	77.9±22.3	72.6±18.9	77.7±16.8	79.0±15.6	0.002	0.78
Burden of kidney disease	34.1±23.3	38.1±23.9	41.8±27.1	33.5±24.7	37.5±24.7	38.2±22.9	0.001	0.45
Work status	69.4±40.8	70.9±41.9	70.9±41.0	76.1±37.3	74.6±37.3	75.4±37.3	0.66	0.58
Cognitive function	85.0±18.6	87.8±16.7	88.2±16.2	86.1±16.6	88.6±13.9	88.1±15.2	0.07	0.82
Quality of social interaction	83.3±19.2	85.9±17.4	86.9±15.8	84.6±16.3	85.5±15.1	86.4±14.7	0.12	0.68
Sleep	64.6±19.8	63.6±18.4	64.7±17.5	65.5±15.6	67.6±17.1	66.5±15.2	0.39	0.52
(non-health related)								
Social support	75.1±18.5	73.3±23.7	78.1±21.0	72.7±22.3	71.4±22.3	70.5±25.4	0.42	0.18
Dialysis staff encouragement	76.5±17.4	77.5±20.2	81.8±16.8	70.5±21.1	70.0±24.2	70.5±22.6	0.08	0.32
Patient satisfaction	83.0±13.5	78.4±19.2	80.7±14.7	77.1±17.0	80.1±16.9	78.9±19.2	0.67	0.42

Acute fatigue was evaluated with a VAS. Chronic fatigue-related symptoms were evaluated with a 64-item questionnaire (2). QOL was evaluated via the KDQOL-SF version 1.3. All variables were tested via 2-way mixed model repeated measure analysis.

### Changes in fatigue-related quantitative measures

**Fig 2:**
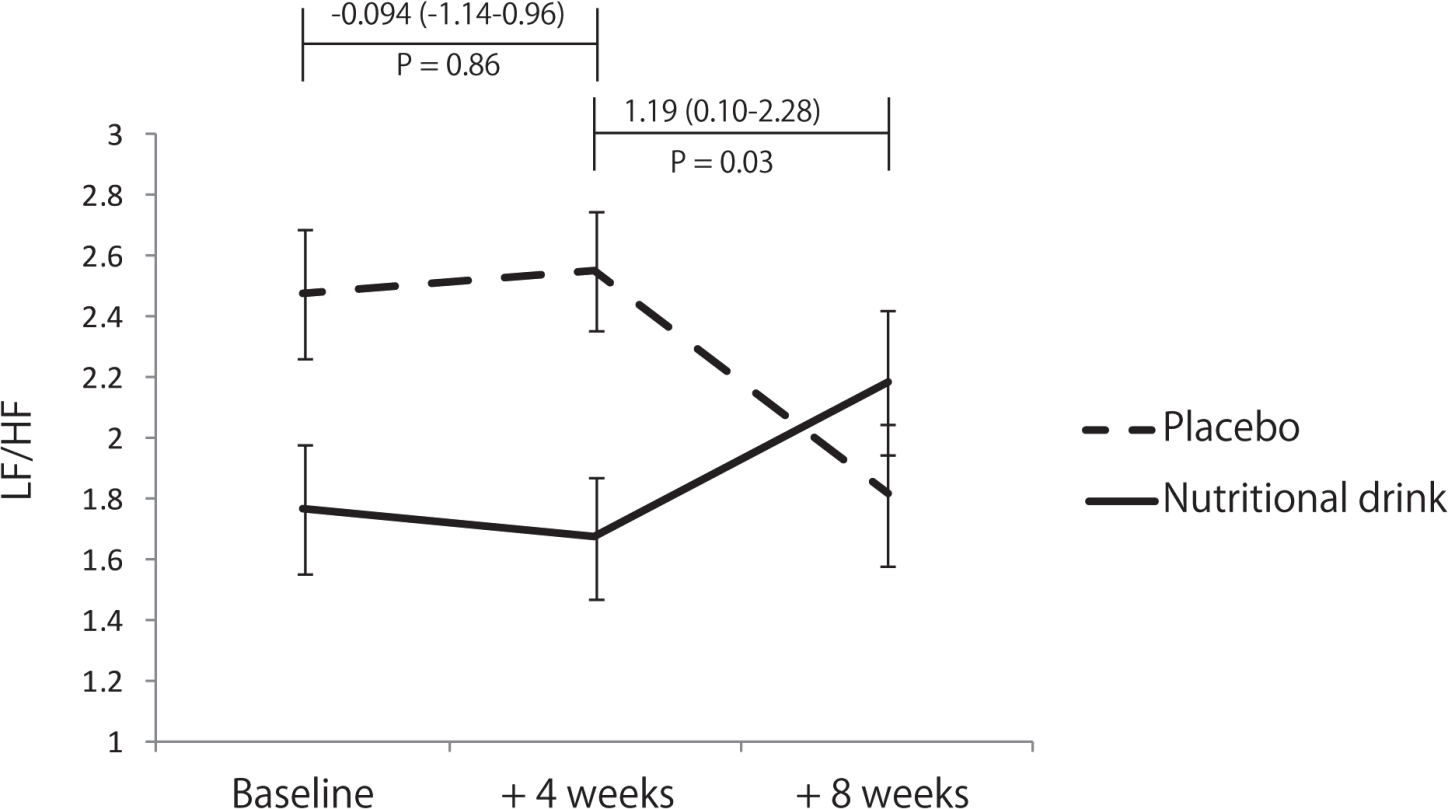
Changes in HRV; LF/HF value (0–4 and 0–12 weeks). LF/HF ratio. Error bars represent the standard error of the mean. Differences of changes in LF/HF ratio between the nutritional drink and placebo groups were determined via the Mann–Whitney test. The baseline LF/FH did not differ significantly between the groups (Z = −0.87, P = 0.39).

**Fig 3:**
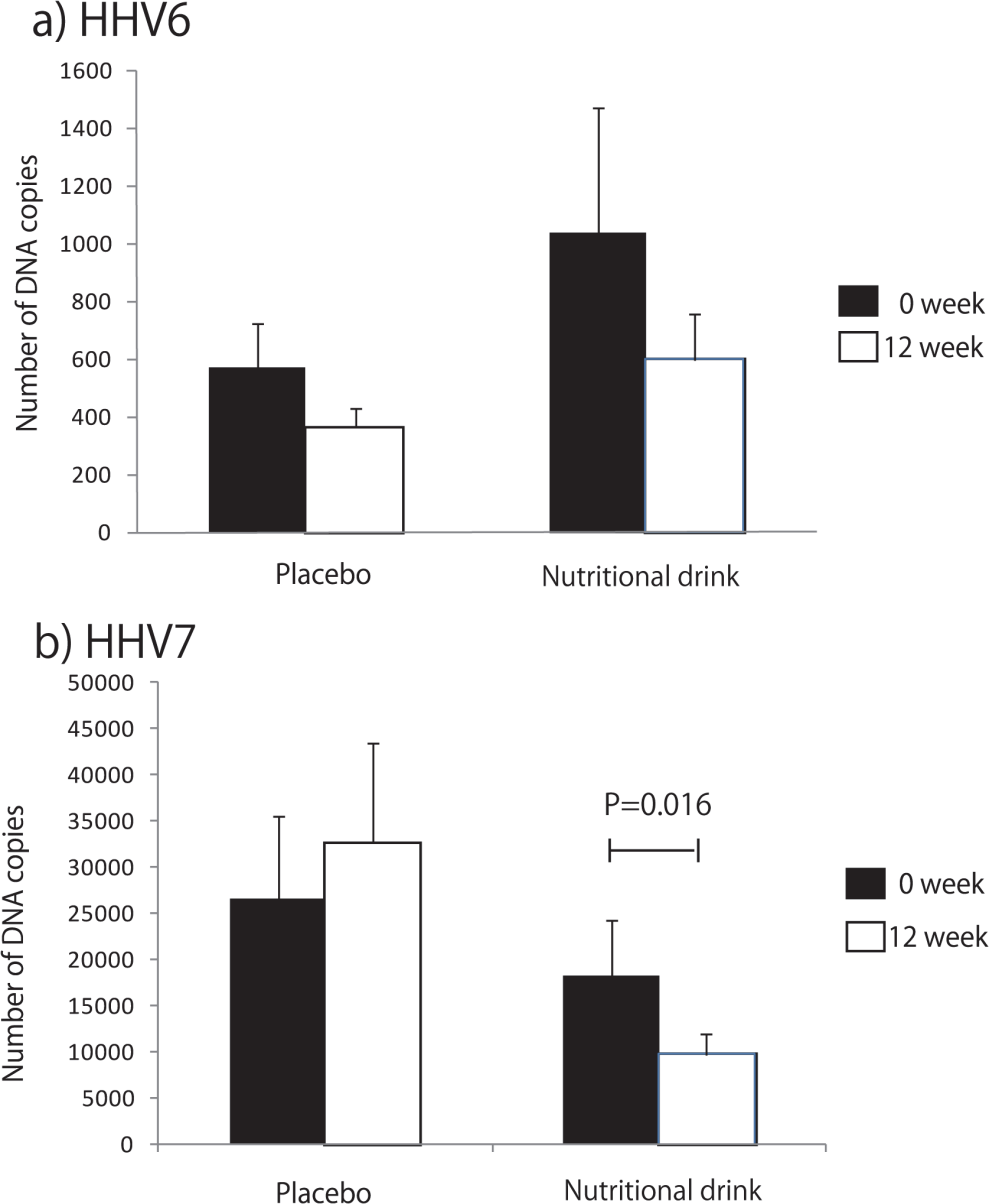
Changes in HHV6 and 7 reactivation following nutritional supplementation in patients with ESRD (0–12 weeks). Differences of changes in the number of copies of (a) HHV6 and (b) HHV7 DNA were determined via the Mann–Whitney test by treatment groups, placebo and nutritional drink (HHV6, Z = −6.08, P = 0.54 and Z = −0.18, P = 0.86 and HHV7, Z = −0.62, P = 0.54 and Z = −2.43, P = 0.016, respectively). We performed the statistical analysis for the samples that exceeded the detection limits, thereafter, the number of participants with these parameters were smaller than in the total samples (HHV6, 23 in the placebo and 22 in the nutritional drink groups, and HHV7, 50 in the placebo and 60 in the nutritional drink groups). Error bars represent the standard error of the mean.

The baseline levels of potential fatigue-related biomarkers, CVa-a, LF/HF, HHV6, HHV7, ACTH, cortisol, and α-MSH between the placebo and nutritional drink groups did not differ significantly. The effects of nutritional drink versus placebo on autonomic and endocrine function, which are potential markers for chronic fatigue, are presented in Table 3 and Fig. 2. Changes in CVa-a (%) during weeks 0–4 and 0–12 were not significantly different between the placebo and nutritional drink groups (placebo; week 0, 3.82 ± 3.92, week 4, 3.54 ± 3.07, and week 12, 3.43 ± 3.66, nutritional drink; week 0, 3.54 ± 3.42, week 4, 3.44 ± 3.48, and week 12, 3.44 ± 3.03). However, changes in LF/HF (0–12 weeks) were significantly higher in the nutritional drink than placebo group, although the changes in LF/HF (0–4 weeks) did not differ between the groups (Fig. 2). The numbers of DNA copies of HHV6 and 7 following nutritional supplementation are presented in Fig. 3. There was a tendency towards but no significant decrease in HHV6 over the 12-week period; however, there was a significant decrease in HHV7 reactivation following nutritional supplementation (P = 0.016) (Fig. 3). The changes in the ACTH–cortisol axis and serum α-MSH were not significantly different between the groups (Table 3). With the exception of serum glucose, changes in any of the hematological and biochemical parameters were not significantly different between the placebo and nutritional drink groups (Table 3). Unexpectedly, nutritional drink decreased serum glucose levels during the trial.

**Table 3:** Changes in HRV, endocrine function and laboratory data throughout the clinical trial.

	Allocated groups						
	Placebo drink			Nutritional drink			*P*-value (time and treatment)
	Week 0	Week 4	Week 12	Week 0	Week 4	Week 12	
Endocrine factors							
ACTH (pg/mL)	51.4±27.5	-	49.8±25.0	50.4±23.9	-	51.3±22.2	0.21
Cortisol (μg/mL)	7.81±2.46	-	8.63±2.76	8.53±2.78	-	9.17±2.74	0.65
α-MSH (pg/mL)	11.9±4.45	-	11.9±5.55	12.7±5.51	-	12.5±6.60	0.76
Laboratory data							
White blood cells (×10^3^/μL)	5.98±1.89	5.95±1.82	5.96±2.17	5.96±1.88	5.90±1.85	6.08±1.75	0.73
Hemoglobin (g/dL)	10.3±1.00	10.4±0.93	10.6±1.06	10.3±0.90	10.3±0.95	10.5±0.99	0.61
Platelets (×10^3^/μL)	172.2±55.6	180.5±57.4	173.3±53.8	167.9±51.0	174.7±51.3	176.7±51.1	0.25
Albumin (g/dL)	3.92±0.27	3.91±0.26	3.91±0.31	3.90±0.31	3.89±0.30	3.88±0.32	0.91
AST (IU/L)	12.9±7.52	12.0±5.75	12.1±6.54	14.1±8.68	14.0±6.88	14.0±6.48	0.48
ALT (IU/L)	12.7±11.4	11.8±6.70	11.8±7.20	12.7±79.0	13.8±.06	13.5±6.81	0.12
LDH (IU/L)	171.3±39.4	171.3±37.8	175.6±40.2	177.8±34.7	178.9±35.9	180.5±32.0	0.68
Creatinine (mg/dL)	12.9±2.33	13.0±2.33	13.2±2.44	12.7±2.20	12.8±2.24	13.0±2.33	0.48
Na (mg/dL)	139.6±3.17	138.8±3.25	138.8±.85	140.0±2.89	139.2±2.93	139.1±2.71	0.92
K (mg/dL)	5.09±0.74	5.01±0.69	4.97±0.74	5.15±0.72	5.13±0.78	4.96±0.69	0.19
Ca (mg/dL)	9.00±0.78	9.02±0.83	9.10±0.84	8.94±0.81	9.00±0.82	9.08±0.80	0.79
P (mg/dL)	5.78±1.31	5.87±1.70	5.48±1.42	5.89±1.46	5.83±1.63	5.78±.27	0.23
Fe (mg/dL)	62.9±24.7	64.5±29.2	63.0±27.3	57.7±25.2	57.3±22.9	63.0±31.0	0.38
Glucose (mg/dL)	102.6±34.2	102.6±37.8	110.1±47.0	117.5±64.7	110.4±50.1	105.9±41.0	0.003
Cholesterol (mg/dL)	151.5±33.2	149.7±34.1	150.1±31.1	154.0±33.1	150.7±31.4	151.2±29.8	0.83
HDL cholesterol (mg/dL)	48.3±14.3	47.2±14.8	45.2±14.7	47.1±15.4	46.6±14.6	45.5±14.6	0.26
Triglyceride (mg/dL)	114.2±75.5	120.7±84.5	135.2±85.4	127.4±83.2	126.8±78.6	129.6±71.7	0.16
CRP (mg/dL)	0.19±0.35	0.33±1.11	0.42±0.48	0.25±0.48	0.33±0.76	0.32±0.66	0.77

All variables were tested via a 2-way mixed model repeated measure analysis.

Conversion factors for units: ACTH pg/mL to pmol/L ×0.22, cortisol μg/mL to nmol/L ×27.59, αMSH pg/mL to pmol/L ×0.60, white blood cell count ×10^3^/μL to 10^9^/L, hemoglobin in g/dl to g/L ×10, platelets, albumin in g/dl to g/L ×10, creatinine in mg/dL to μmol/L ×76.26, Na in mg/dL to mmol/L ×88.4, K in mg/dL to mmol/L ×0.4349, Ca in mg/dL to mmol/L ×0.2495, P in mg/dL to mmol/L ×0.3228, Fe in mg/dL to mmol/L ×0.1791, glucose in mg/dL to mmol/L ×0.0551, cholesterol and HDL cholesterol in ng/dl to mmol/L ×0.02586, triglyceride in mg/dL to mmol/L ×0.01129, CRP in mg/dl to ng/L ×100. No conversion necessary for AST, ALT and LDH in IU/L.

ACTH, adrenocorticotropic hormone; ALT, alanine aminotransferase; AST, aspartate aminotransferase; CRP, C-reactive protein; HDL, high-density lipoprotein; LDH, lactate dehydrogenase; MSH, melanocyte-stimulating hormone.

### Safety and hospitalization

In the nutritional drink group, one participant reported increased blood pressure, one complained of dizziness, one complained of insomnia, one reported nausea, and two had diarrhea. One participant in each group had cramp in the lower leg. In the placebo group, one participant reported increased glucose level, two felt sick, and one complained of stomach discomfort. One participant developed sudden hearing loss and was prescribed any vitamins. Two participants were hospitalized in the placebo group. The safety monitoring board confirmed no serious adverse events, and hospitalization was determined relating to the study intervention.

## Discussion

The aim of the present study was to assess the effects of nutritional supplementation during dialysis on various fatigue-related subjective and objective measures. We found that nutritional supplementation was associated with favorable changes in autonomic and immune function.

Importantly, nutritional problems are one of the risk factors in the pathogenesis of cardiovascular disease in ESRD patients [22], and could be associated with impaired autonomic function as well. However, the effects of nutritional supplementation on cardiovascular functions in ESRD have been rarely reported. Niacin supplementation helps to induce anti-inflammatory systems in the remnant kidney [23]. Folic acid treatment possibly decreases the risk of cardiovascular disease in chronic renal failure [24]. Low plasma vitamin C levels in ESRD patients with any comorbidity might carry a high mortality risk [25]. Carnitine treatment has been shown to have cardiovascular benefits, including a decrease in the incidence of arrhythmias, preservation of mechanical function, and suppression of left ventricular hypertrophy [26]. These potential beneficial effects of carnitine may be attributable to modulation of myocardial β-oxidation [27]. To the best of our knowledge, there have been no reports on the effects of nutritional supplementation or certain supplements on autonomic nervous activity in ESRD patients. However, there are reports on improvement of autonomic activity following nutritional supplementation in healthy individuals [28–30]. l-Arginine and vitamin C have been shown to modulate substantially sympathetic-mediated peripheral vascular resistance [28]. Vitamin C intake also has the potential to improve sympathetic dysfunction resulting from injury by excessive oxidative stress after myocardial infarction [29]. Last, CoQ10 may increase fat oxidation by augmenting autonomic nervous activity during low intensity exercise in healthy individuals [30]. Hemodialysis patients show sympathetic dysfunction, that is, hypotension during hemodialysis [31]. These observations are considered to be predictive for cardiac damage and sudden death. Our observations suggest that nutritional supplementation improves sympathetic activity in ESRD patients, without a significant effect on total autonomic activity. CVa-a did not change throughout the study. Long-term supplementation might be needed to further improve other autonomic parameters.

LF/HF ration in healthy individuals is significantly and positively associated with fatigue score [32]. Moreover, LF/HF ratio in ESRD patients was significantly and positively associated with several components of QOL, including physical functioning and role-emotional, independent of other clinical parameters [8]. However, in the present trial, subjective symptoms, such as fatigue and QOL, improved in both the placebo and nutritional drink groups, suggesting that subjective symptoms might be markedly influenced by a placebo effect. l-Carnitine significantly improved fatigue in ESRD, after 12 and 24 weeks of treatment, compared with placebo, using the primary analysis [15]. Compared with our study, the dose of carnitine in that study was twice that in our study, and the intravenous supplementation methods differed from the oral supplementation in our study.

There have been no reports on the effects of nutritional supplementation or certain supplements on immune dysfunction, or the prevention of infection in ESRD patients. Our data clearly showed that nutritional supplementation suppressed HHV7 but not HHV6 reactivation. Human HHV6 and HHV7 are more common in patients with chronic fatigue syndrome [11]. The detailed underlying mechanisms regulating HHV6 and HHV7 reactivation have not been well clarified. In our preliminary observations, HHV7 reactivation was higher in patients with ESRD than in healthy individuals, while HHV6 DNA numbers in saliva did not differ markedly in patients undergoing hemodialysis (unpublished observation). Thus, HHV7 reactivation might represent fatigue or altered immune function more efficiently than HHV6 in ESRD patients. Although further studies are apparently necessary to unveil the mechanisms of our observations, our results do emphasize the importance of nutritional supplementation on fatigue or immunological function in ESRD patients.

There were several limitations to our study. First, most of the outcome parameters related to fatigue are still not widely utilized; therefore, we classified our trial as a pilot study to survey the significance of several candidate fatigue measures. Second, there have been no similar trials before; thus, we could not estimate the numbers of participants required to achieve sufficient power for analysis, however sample size analysis for the LF/HF was performed at the baseline patients after the study, and 143 patients were needed set at alpha error 0.05 and beta error 0.9, therefore our trial was enough power for the statistical analysis. Third, we used the combination of multiple nutrients for nutritional drinks; therefore, the significance of each of the nutritional components could not be determined. Despite these limitations, we believe our study is an important first step to understanding further the role of nutritional support for fatigue and related pathophysiological conditions in ESRD patients.

In summary, this is believed to be the first randomized controlled trial to show that nutritional supplementation is potentially beneficial for autonomic nervous dysfunction and immunological alteration in ESRD patients. Further long-term controlled studies are needed to evaluate the benefits of nutritional supplementation for clinical outcomes in ESRD patients.

## Supporting Information

S1 CONSORT Checklist (DOC)

S1 Protocol (DOCX)
