# Supplementary material for: Effects of Nutritional Supplementation on Fatigue, and Autonomic and Immune Dysfunction in Patients with End-Stage Renal Disease: A Randomized, Double-Blind, Placebo-Controlled, Multicenter Trial
Source: PLoS One. 2015 Mar 6;10(3):e0119578. doi: 10.1371/journal.pone.0119578 (PMC4352065; doi:10.1371/journal.pone.0119578)
Supplement: S1 Protocol — (DOCX) [file pone.0119578.s002.docx]

**Protocol:** Efficacy of the nutrients supplement “AMP01” for the fatigue of the hemodialysis patients

**Principal Investigator:** Yoshiki Nishizawa, Osaka City University, 545-8585, Osaka

**Collaborating hospitals and regional principal investigators:**

Tsutomu Tabata, Inoue hospital,

Mikio Okamura, Ohno Memorial hospital,

Tomoyuki Yamakawa, Shirasagi hospital,

Shigeki Okada, Okada Clinic

The first application date of the protocol: 06/November/2007 The protocol was fixed.

(The approval date: 13/December/2007)

The second approval date of the protocol: 07/April/2008 (The Approval date: 15/May/2008), The minor variation from the original protocol.

The third approval date of the protocol: 30/April/2008 (The approval date: 12/June /2008), Okada Clinic was added to the collaborators of the trial.

**1 The background of the study**

The survey for 1122 hemodialysis patients at 2005 suggests that prevalence of fatigue is higher in these patients than healthy controls (Japan Dialysis Conference 2007). These patients represent higher scores for symptoms of depression and autonomic function. It has been well known that nutrients such as vitamins and carnitine are lost during hemodialysis session.

**2 Purpose**: To investigate an efficacy of supplementation of nutrients with “AMP01” for the fatigue of the hemodialysis patients by a randomized double-blind placebo-controlled study.

**3 Test food**

**AMP01**, the nutritional 50ml drink

One brown bottle (50 ml) contains carnitine 500mg, CoQ10 30mg, oligosaccharide 5g, zinc 8mg, vitamin B1 10mg, vitamin B2 1.8mg, niacin 15mg, vitamin B6 10mg, vitamin B12 30microg, folic acid 500microg, vitamin C 60mg in slightly yellow liquid.

**Placebo**, One brown bottle (50 ml) contains drink which appearance and flavour are the same as the test drink.

**4 Methods**

**4.1 Recruitment of patients**:

**4.1.1 Inclusion criteria:**

(1) Patients who are equal or more than 30-year-old and less than 70 year-old at the time of getting the Informed consent
(2) Patients undergoing regular afternoon or night hemodialysis
(3) Hemodialysis duration is more than one year
(4) Patients who receive hemodialysis three times a week
(5) Patients who are capable of understanding the study, and those who can give written informed consents
(6) Patients who can adhere instructions

**4.1.2 Exclusion criteria:**

(1) Patients who are taking nutrients supplements. If the patients can stop taking the supplements two weeks before the study, the patients do not meet exclusion criteria.
(2) Patients who receive vitamin compound (except Vitamin D) for treatment, or take OTC drugs for vitamin supplementation. If the patients can stop the treatment two weeks before the study, the patients do not meet exclusion criteria.
(3) During the pregnancy or nursing, or patients to be pregnant during the study period
(4) patients who participated in other clinical trials for food or drug within the past three months
(5)Patients with allergy against constituents of the supplement
(6) Others judged by physicians

**4.2. Design**: Parallel, Randomized, Double blind, Placebo-controlled trial.

**4.3. Outline of the trial:** The treatment period was 12 weeks, with a midpoint assessment after 4 weeks from individual enrolment.

**4.4 Informed consent**

All participants consented to participate in the study before enrolment by written form. PI and co-PI determine patients who are eligible for participants candidate of the study. The clinical research coordinators representing for PI or co-PI explain the detail of the trial to potential participants.

**4.5 Registration:** The data centre will be in charge of collecting and managing clinical data. The data centre will handle site registration, participant registration and allocation and monitor data entry.

**4.6 Allocation to treatments:**

(1)Randomization was by means of random number table (1:1), stratified by gender, age, and institutions.

(2) An originally assigned code numbers for each test food and placebo were kept in each enclosed envelop within the data enter. Regional hospital PI control individual assigned code numbers which can be opened at the occurring acute serious adverse events (SAE).

**4.7 Taking test food**

Participants take one bottle of placebo or AMP01 three times a week just after hemodialysis sessions for 12 weeks

**4.8 Control of test food**

The central controller of test food at Osaka City University (OCU) Hospital preserve and control the test food depended on the manual documents after the food are delivered to the OCU hospital from the funding company.

Regional controllers of test food at individual clinic or hospitals also preserve and control the test food depended on the manual documents after the food are delivered to the clinic or hospitals from the OCU hospital.

**4.9 The schedule of the study** Acceleration plethysmogram, fatigue markers (alpha-MSH, ACTH, cortisol, HHV6, 7), questionnaires for acute and chronic fatigue, questionnaires for QOL (KDQOL-SF), clinical characteristics, and laboratory data

Table 1: Measures used and time of measurement

|  | Before the trial | Baseline | 4-weeks | 12-weeks  (End of the drinking) |
| --- | --- | --- | --- | --- |
| Questionnaire for acute fatigue |  | × | × | × |
| Questionnaire for chronic fatigue |  | × | × | × |
| questionnaires for QOL (KDQOL-SF) |  | × | × | × |
| Acceleration plethysmogram |  | × | × | × |
| alpha-MSH |  | × |  | × |
| ACTH |  | × |  | × |
| cortisol |  | × |  | × |
| Saliva HHV6 |  | × | * | × |
| Saliva HHV7 |  | × | * | × |
| Laboratory data |  | × | × | × |
| Weight, height and vital sign |  | × | × | × |
| Clinical characteristic data | × |  |  |  |

* measured if necessary after the analyses of 12-week samples

**4.10 Measurements**

The demographic and clinical data were recorded, as well as the use of medication and lifestyle parameters.

Non-fasting blood and saliva samples were collected prior to the first dialysis session (after the weekend) at week 0 and 12 of the trial for the determination of blood counts and clinical chemical, lipid, and inflammation parameters. As potential biomarkers of fatigue, serum adrenocorticotropic hormone (ACTH), measured via an immunoradiometric assay, and serum cortisol and α-melanocyte stimulating hormone (α-MSH) measured by radioimmunoassay were determined. Virus DNA copies of HHV6 and 7 are measured at Tokyo Jikei Medical College. All laboratory analyses, except HHV6 and 7, and α-MSH, were performed in the central laboratory of Mitsubishi Chemical Medience Corporation, Ltd. (Japan).

Fatigue and QOL were measured at weeks 0, 4 and 12 of the trial. Subjects were asked to rate how often they experienced the symptoms of fatigue in a recent week using a Likert scale (0–4). Eight factors were calculated as a part of the principal factor analysis with a proximal rotation. They were as follows: 1) fatigue, 2) anxiety and depression, 3) loss of attention and memory, 4) pain, 5) overwork, 6) autonomic imbalance, 7) sleep problems, and 8) infection. The acute fatigue scale associated with hemodialysis was evaluated with a Visual Analog Scale (VAS). QOL was evaluated via the Kidney Disease Quality of Life (KDQOL-SF^TM^) questionnaire. Heart rate variability (HRV) of each participant was measured and analyzed with an acceleration plethysmography (APG) system (ARTETD, Umedica Inc., Osaka, Japan).

**4.11 Target sample size:**200 (100 in each arm)

5 **Ethical Aspects**

Procedures for informed consent

Prior to the trial participation, the trial PI must explain the following items using the written consent form and make sure that participant has understood the content of the trial well. Written informed consent will only then be obtained from the participants.

1) Clinical status of participant

2) The purpose of the trial

3) Name of the principal investigator

4) The trial treatment(s) and the trial procedures

5) The reasonably expected benefits

6) The reasonably foreseeable risks

7) The alternative procedure(s) or course(s) of treatment that may available to the subjects

8) The anticipated expenses and prorated payment to the subject for participating in the trial

9) The compensation and/or treatment available to the subject

10) Intellectual property right policy

11) There is no disadvantage if the subject does not participate in the trial or stops participating in the trial

12) Participants can refuse or withdraw from the trial, at any time, without penalty

13) Privacy will be maintained and protected

14) Fundings source for the trial, Conflict of the interest, Contact address and method when the participant wants more information about the clinical trial or when he/she feels unwell

**6 Rules for dropping out from the trial**

If the participant meets any one of the following conditions, the trial physician will stop the protocol treatment at his/her discretion.

1) The participant wishes to stop the protocol treatment

2) The participant is found not to meet criteria after the registration

3) The trial physician judges that the risk outweighs benefit in continuing the protocol treatment even no serious adverse events (SAE) is reported

4) The participant needs admission to a hospital with suffering complications

5) The trial physician judges that it is difficult to continue the protocol treatment because of emergence of SAE as defined below

6) The compliance status will be apparently poor (less than 80% for all intakes or over 120% for all intakes)

7) If the trial will stop for some reasons

8) The participant does not adhere to the rule

9) The trial physician judges that it is inappropriate to continue the protocol treatment for any other reason

**6.1 Adherence for the participant**

1) Not intake any other supplements except the test drink during the trial

2) Not intake any other drugs for vitamins and other nutritional supplementation except the test drink during the trial

**7 Reporting of adverse events and protection of participants**

**7.1 Definition of adverse events**

An adverse event is defined as any unwanted or unintended sign (including laboratory exams), symptom or disease seen in participants of the trail, regardless of the causal relationship with the study intervention.

**7.2 Managing for adverse events**

The trial physician makes appropriate treatment and reports documents for the event soon after the adverse events. If the physician judges to stop the trial, he/she must inform the participant about this and its treatment for the events.

**8 Statistical Analyses**

Sample size: This is the pilot study and is a feasibility study and needs no sample size calculation. The target sample size is 200. The outcome of the study was the changes in the acute and chronic fatigue scales, QOL, levels of serum ACTH, cortisol and α-MSH, HHV6 and 7, and autonomic function, as determined by HRV between testing food groups. P<0.05 is defined as the statistical significance.

**9 Outcome assessments for efficacy**

Acceleration plethysmogram, fatigue markers (alpha-MSH, ACTH, cortisol, HHV6, 7), questionnaires for acute and chronic fatigue, and questionnaires for QOL (KDQOL-SF)

**10 Outcome assessments for safety**

Laboratory data (biological and clinical), SAE

**11 Discontinuation of the trial**

The PI considers discontinuation of the trial when,

(1) serious concerns for the safety of the test food arise

(2) ethical committee advise to change the protocol

, and reports to the director of the Hospital as soon as possible, if decide to discontinue.

**12 Study Period:** January/2008-August/2008, The first enrolment of the study: May/2008

**13 Data handling:** The data center will be in charge of collecting and managing all data and information. The data centre will manage quality control of data, and prepare datasets for statistical analyses.

**14 Regulations to be adhered to**

**14.1 Approval by the IRB**

The protocol is to be approved by the Ethics Committee of Osaka City University Graduate School of Medicine, and of regional Hospitals. All the researchers participating in the trial will place the participants ‘safety and human rights above everything else and will adhered by the study protocol and the study manual so long as they do not undermine their safety and human rights.

**14.2 Adherence to the study protocol and study manual**

All the researchers participating in the trial will abide by the Declaration of Helsinki and its amendments as well as the Ethics Guideline for epidemiological study ( Ministry of Health, Labour and Welfare).

**14.3 Protection of privacy**

All the researchers and outsourcers of this trial must strictly protect personal information of the participants in adherence with Ethics Guidelines for Epidemiological Research. Each trial site will collect information in anonymized and linkable format. The linking information for the participants is strictly managed at each trial site or at the data center.

**15 Compensation Insurance and payment**

All participants receive 15,000YEN for expenses including travel cost. If there might happen some health hazards which could not be denied its causal association between test drink and health hazards, the company covered with it.

**16 Publication policy**

The results of the trial will be published without any hazards to privacy of participants

**17 Research Organization**

**Steering Committee**

The Steering Committee hold meetings before and after the trial.

**Principal Investigator:** Yoshiki Nishizawa, Osaka City University, 545-8585, Osaka

**Collaborating hospitals and regional principal investigators:**

Tsutomu Tabata, Inoue hospital,

Mikio Okamura, Ohno Memorial hospital,

Tomoyuki Yamakawa, Shirasagi hospital,

Shigeki Okada, Okada Clinic

**Sources of funding**

Asahi Kasei Kuraray Medical, Co..LTD, Japan

**The chief allocator**

Mitsuru Fukui, Osaka City University, Department of Statistical Medicine

**Trial statistician**

Sanae Fukuda, PhD., Osaka City University, Department of Physiology

**Site manager**

Hidekiti Tokai, Osaka City University Hospital, Pharmacists (now at Osaka Tetsudo Hospital)

**Date manager**

Hisako Fujii, Osaka City University Hospital, Pharmacists

**Protocol committee:**

Chief investigator: Hidenori Koyama, Osaka City University (now at Hyogo Medical College)

Yasuyoshi Watanabe , Osaka City University(now at RIKEN)

Yoshinobu Hirayama, Osaka City University Hospital (now at Ritsumeican University)

Sumio Hirata, Kumamoto University

Sanae Fukuda, Osaka City University (now at RIKEN)

**Other collaborators:**

Hiroshi Kiyama, Osaka City University (now at Nagoya University)

Kazuhiro Kondo, Tokyo Jikei Medical College

**17 Data management**

Data Management

The data management will be done by the data center. The electronic data is anonymized in a linkable record, and the participants’ names and ID numbers will be recorded only on non-electronic media (e.g. paper notebook) and kept at each trial site.

The datacenter will progress of all the entered participants and will contact the CRC or the trial physician should any doubt arise.

**18 Conflict of interest**

This study is performed by the fund which is provided by the Asahi Kasei Kuraray Medical, Co..LTD, Japan.
